# Chlorido[2,2′-[1,2-phenyl­enebis(nitrilo­methanylyl­idyne)]diphenolato-κ^4^ *O*,*N*,*N*′,*O*′]manganese(III) methanol monosolvate

**DOI:** 10.1107/S1600536813016450

**Published:** 2013-06-19

**Authors:** Hui Lin, Jian-Gang Wang, Hua-Tian Shi, Qun Chen, Qian-Feng Zhang

**Affiliations:** a Institute of Molecular Engineering and Applied Chemsitry, Anhui University of Technology, Ma’anshan, Anhui 243002, People’s Republic of China; b Department of Applied Chemistry, School of Petrochemical Engineering, Changzhou University, Jiangsu 213164, People’s Republic of China

## Abstract

In the title complex, [Mn(C_20_H_14_N_2_O_2_)Cl]·CH_3_OH, the central Mn^III^ atom displays a distorted square-pyramidal coordination by two N and two O atoms from the tetradentate 2,2′-[1,2-phenyl­enebis(nitrilo­methanylyl­idyne)]diphenolate ligand and one chloride ligand. The Mn^III^ atom is 0.525 (4) Å out of the square basal N_2_O_2_ least-squares plane. The complex mol­ecule is hydrogen bonded to the methanol solvent mol­ecule.

## Related literature

For background to manganese and manganese–salen complexes, see: Law *et al.* (1998 ▶); Lenoble *et al.* (1998 ▶); Horner *et al.* (1999 ▶); Asada *et al.* (2000 ▶); Dubois *et al.* (2003 ▶); Gultneh *et al.* (2003 ▶); Mitra *et al.* (2006 ▶). For related structures, see: Pecoraro & Butler (1986 ▶); Dang *et al.* (2005 ▶); Martínez *et al.* (2002 ▶); Panja *et al.* (2003 ▶).
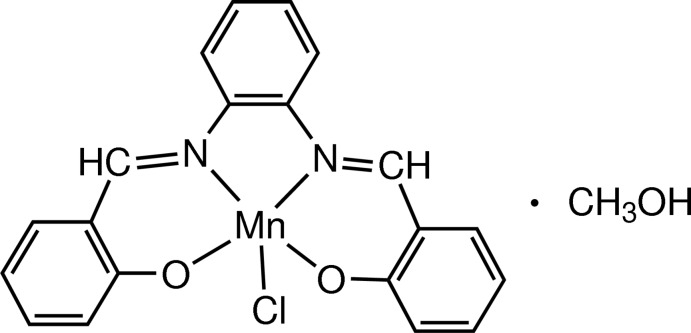


## Experimental

### 

#### Crystal data

[Mn(C_20_H_14_N_2_O_2_)Cl]·CH_4_O
*M* *_r_* = 436.76
Triclinic, 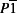

*a* = 7.4251 (2) Å
*b* = 9.8341 (2) Å
*c* = 13.3035 (3) Å
α = 78.803 (1)°
β = 83.305 (2)°
γ = 86.344 (2)°
*V* = 945.58 (4) Å^3^
*Z* = 2
Mo *K*α radiation
μ = 0.87 mm^−1^
*T* = 296 K
0.24 × 0.17 × 0.13 mm

#### Data collection

Bruker SMART APEXII CCD area-detector diffractometer
Absorption correction: multi-scan (*SADABS*; Bruker, 2001 ▶) *T*_min_ = 0.819, *T*_max_ = 0.896
17011 measured reflections
4302 independent reflections
3311 reflections with *I* > 2σ(*I*)
*R*_int_ = 0.032

#### Refinement

*R*[*F* ^2^ > 2σ(*F* ^2^)] = 0.035
*wR*(*F* ^2^) = 0.086
*S* = 1.02
4302 reflections
255 parameters
1 restraint
H-atom parameters constrained
Δρ_max_ = 0.33 e Å^−3^
Δρ_min_ = −0.40 e Å^−3^

### 

Data collection: *APEX2* (Bruker, 2007 ▶); cell refinement: *SAINT* (Bruker, 2007 ▶); data reduction: *SAINT*; program(s) used to solve structure: *SHELXS97* (Sheldrick, 2008 ▶); program(s) used to refine structure: *SHELXL97* (Sheldrick, 2008 ▶); molecular graphics: *SHELXTL* (Sheldrick, 2008 ▶); software used to prepare material for publication: *SHELXTL*.

## Supplementary Material

Crystal structure: contains datablock(s) I, global. DOI: 10.1107/S1600536813016450/vn2073sup1.cif

Structure factors: contains datablock(s) I. DOI: 10.1107/S1600536813016450/vn2073Isup2.hkl

Additional supplementary materials:  crystallographic information; 3D view; checkCIF report

## Figures and Tables

**Table 1:** Hydrogen-bond geometry (Å, °)

*D*—H⋯*A*	*D*—H	H⋯*A*	*D*⋯*A*	*D*—H⋯*A*
O1*S*—H1*S*⋯O1^i^	0.87	2.19	2.999 (3)	154

Symmetry code: (i) 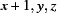.

**Fig. 1.**
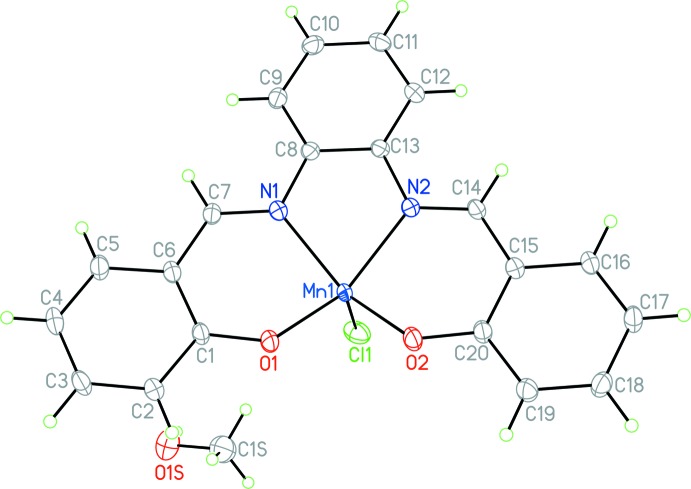
The structure of [MnIII(salen)Cl] (salen = N,N'-bis(salicylideneiminato)- benzene); displacement ellipsoids at the 50% probability level.

**Fig. 2.**
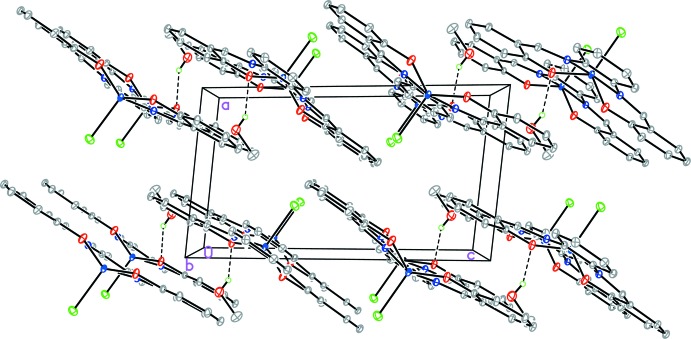
Packing diagram of the title complex in a unit cell. Dash lines denote the intermolecular (CH3)O—H···O(salen) hydrogen bonds.

## supplementary crystallographic information

### Comment

The coordination chemistry of manganese complexes has been the subject of extensive investigation in the past several decades. Most of the studies have aimed to understand the role of manganese in many metallo-enzymes in terms of structure- property relationships (Dubois *et al.*, 2003; Horner *et al.*, 1999). Studies of high oxidation state complexes are of special importance because of their potential uses as oxidizing agents, catalysts and electro-catalysts, for the oxidation of compounds such as alcohols, esters and water (Gultneh *et al.*, 2003). Of particular interest is the Schiff base complexes of manganese(III) which have been considered to be the simplest models for the reactivity of oxygen-evolving center (OEC) active site of mangano-enzymes (Law *et al.*, 1998). The typical [Mn^III^(salen)X] (X = Cl, Br, I) complexes (salen = *N*,*N*'-bis(salicylideneiminato)ethylene) have been prepared and are soluble in aqueous and methanolic solutions (Mitra *et al.*, 2006). Series of monochloro- and dichloro-manganese(IV) complexes along with acetatomanganese(III) complexes with the salen ligands have been previously synthesized from the mixed aqueous-ethanol or -acetonitrile solutions (Asada *et al.*, 2000; Lenoble *et al.*, 1998; Pecoraro & Butler, 1986). In this paper, we report the synthesis of the manganese(III) complex [Mn^III^(salen)Cl].CH_3_OH (salen = *N*,*N*'-bis(salicylideneiminato)benzene) in a mixed aqueous-methanol solution and its structural characterization involving an N_2_O_2_ Schiff base ligand.

The title complex crystallizes in the triclinic *P*-1 space group. The asymmetric unit of the crystal structure consists of the neutral mononuclear complex [Mn^III^(salen)Cl] and one methanol molecule in the lattice. A view of the complex is shown in Fig. 1. In this monomeric complex, the central manganese atom is coordinated by two nitrogen and two oxygen atoms from the salen ligand and one chloride atom. Owing to the presence of the chloride atom, the manganese atom is 0.525 (4) Å above the square basal N_2_O_2_ plane and the geometry around the metal centre may be better described as distorted square-pyramidal. The average Mn—N and Mn—O bond lengths in the title complex are 2.0992 (16) and 1.8942 (14) Å, respectively, which are compared with those in [Mn(salen)Cl(H_2_O)].H_2_O (salen = *N*,*N*'- bis(salicylideneiminato)ethylene) [av. Mn—N = 1.984 (17) Å and av. Mn—O = 1.883 (14) Å] (Panja *et al.*, 2003), [MnCl(salen)(H_2_O)] (salen = 2,2'-[1,2-ethanediylbis- (nitrilomethylidyne)]-diphenolato) [av. Mn—N = 1.980 (5) Å and av. Mn—O = 1.890 (4) Å] (Martínez *et al.*, 2002), and [Mn(L)Cl] (L = *N*,*N*'-bis{4-(diethylamino)- salicylideneiminato}-cyclohexane) [av. Mn—N = 1.986 (12) Å and av. Mn—O = 1.872 (12) Å] (Dang *et al.*, 2005). The Mn—Cl bond length of 2.2276 (7) %A in the title complex is obviously shorter than those in [Mn(salen)Cl(H_2_O)].H_2_O (salen = *N*,*N*'-bis(salicylideneiminato)ethylene) (2.584 (12) %A) (Panja *et al.*, 2003), [MnCl(salen)(H_2_O)] (salen = 2,2'-[1,2-ethanediylbis-(nitrilomethylidyne)]-diphenolato) (2.468 (2) Å) (Martínez *et al.*, 2002), and [Mn(L)Cl] (L = *N*,*N*'-bis{4-(diethylamino)salicylideneiminato}cyclohexane) (2.386 (2) Å) (Dang *et al.*, 2005). The basal bond angles are all approximately close to 90° [O(1)—Mn(1)—O(2), O(1)—Mn(1)—N(1) and O(2)—Mn(1)—N(2) are 91.48 (6)°, 87.99 (6)° and 87.68 (6)°, respectively] except N(1)-Mn(1)-N(2) (76.74 (6)°) which is large smaller than expected (Pecoraro & Butler, 1986). The methanol molecule takes part in one hydrogen-bond involving in H1S with the phenoxo-oxygen O1a from the next unit-cell and the distance O1a···H1S (a: *x* + 1, *y*, *z*) is 2.19 (2) Å (see Fig. 2).

### Experimental

To a solution of the Schiff base ligand (H_2_salen) (158 mg, 0.5 mmol) in methanol (10 mL) was added Et_3_N (101 mg, 1.0 mmol), and then a solution of MnCl_2_.6H_2_O (117 mg, 0.5 mmol) in distilled water (5 mL) was dropwise added to the above methanol solution. The resulting solution was refluxed for 4 h, and then the mixture was filtered and the filtrate was allowed to evaporate slowly, which led to deposition of a brown solid. The solid was collected by filtration, washed with Et_2_O and recrystallized from CH_3_OH-Et_2_O mixture (1:1). Yield: 111 mg, 58 %. Analysis for C_21_H_18_N_2_O_3_ClMn: calcd C 57.75, H 4.15, N 6.41 %; found C 57.63, H 4.12, N 6.37 %.

### Refinement

The structure was solved by direct methods and refined by full-matrix least-squares procedure based on F^2^. All hydrogen atoms on the carbon atoms were placed in geometrically idealized positions and refined isotropically with a riding model for both C-sp^2^ [C—H = 0.93 Å and with *U*_iso_(H) = 1.2*U*_eq_(C)] and C-sp^3^ [C—H = 0.96—0.97 Å and with *U*_iso_(H) = 1.5*U*_eq_(C)], except the hydrogen position H1S involved in a hydrogen bond interaction and which was refined with a distance restraint.

### Crystal data

**Table:**

[Mn(C_20_H_14_N_2_O_2_)Cl]·CH_4_O	*Z* = 2
*M**_r_* = 436.76	*F*(000) = 448
Triclinic, *P*1	*D*_x_ = 1.534 Mg m^−^^3^
Hall symbol: -P 1	Mo *K*α radiation, λ = 0.71073 Å
*a* = 7.4251 (2) Å	Cell parameters from 5729 reflections
*b* = 9.8341 (2) Å	θ = 2.4–26.8°
*c* = 13.3035 (3) Å	µ = 0.87 mm^−^^1^
α = 78.803 (1)°	*T* = 296 K
β = 83.305 (2)°	Block, pink
γ = 86.344 (2)°	0.24 × 0.17 × 0.13 mm
*V* = 945.58 (4) Å^3^	

### Data collection

**Table:**

Bruker SMART APEXII CCD area-detector diffractometer	4302 independent reflections
Radiation source: fine-focus sealed tube	3311 reflections with *I* > 2σ(*I*)
Graphite monochromator	*R*_int_ = 0.032
phi and ω scans	θ_max_ = 27.5°, θ_min_ = 1.6°
Absorption correction: multi-scan (*SADABS*; Bruker, 2001)	*h* = −9→9
*T*_min_ = 0.819, *T*_max_ = 0.896	*k* = −12→12
17011 measured reflections	*l* = −17→17

### Refinement

**Table:**

Refinement on *F*^2^	Primary atom site location: structure-invariant direct methods
Least-squares matrix: full	Secondary atom site location: difference Fourier map
*R*[*F*^2^ > 2σ(*F*^2^)] = 0.035	Hydrogen site location: inferred from neighbouring sites
*wR*(*F*^2^) = 0.086	H-atom parameters constrained
*S* = 1.02	*w* = 1/[σ^2^(*F*_o_^2^) + (0.0419*P*)^2^ + 0.2015*P*] where *P* = (*F*_o_^2^ + 2*F*_c_^2^)/3
4302 reflections	(Δ/σ)_max_ = 0.001
255 parameters	Δρ_max_ = 0.33 e Å^−^^3^
1 restraint	Δρ_min_ = −0.40 e Å^−^^3^

### Special details

**Table:**

Geometry. All esds (except the esd in the dihedral angle between two l.s. planes) are estimated using the full covariance matrix. The cell esds are taken into account individually in the estimation of esds in distances, angles and torsion angles; correlations between esds in cell parameters are only used when they are defined by crystal symmetry. An approximate (isotropic) treatment of cell esds is used for estimating esds involving l.s. planes.
Refinement. Refinement of F^2^ against ALL reflections. The weighted R-factor wR and goodness of fit S are based on F^2^, conventional R-factors R are based on F, with F set to zero for negative F^2^. The threshold expression of F^2^ > 2sigma(F^2^) is used only for calculating R-factors(gt) etc. and is not relevant to the choice of reflections for refinement. R-factors based on F^2^ are statistically about twice as large as those based on F, and R- factors based on ALL data will be even larger.

### Fractional atomic coordinates and isotropic or equivalent isotropic displacement parameters (Å^2^)

**Table:**

	*x*	*y*	*z*	*U*_iso_*/*U*_eq_	
Mn1	0.06050 (4)	0.87475 (3)	0.26112 (2)	0.03227 (10)	
Cl1	0.30457 (9)	0.81214 (7)	0.34473 (5)	0.06368 (18)	
N1	0.1304 (2)	1.06118 (16)	0.16224 (12)	0.0368 (4)	
N2	−0.0670 (2)	1.02686 (16)	0.33991 (11)	0.0345 (4)	
O1	0.0801 (2)	0.79008 (14)	0.14311 (10)	0.0475 (4)	
O2	−0.1221 (2)	0.75440 (15)	0.32907 (11)	0.0522 (4)	
C1	0.1710 (3)	0.8242 (2)	0.05065 (14)	0.0388 (4)	
C2	0.2021 (3)	0.7250 (2)	−0.01285 (16)	0.0479 (5)	
H2	0.1588	0.6364	0.0104	0.057*	
C3	0.2960 (3)	0.7574 (3)	−0.10922 (17)	0.0535 (6)	
H3	0.3169	0.6897	−0.1498	0.064*	
C4	0.3601 (3)	0.8889 (3)	−0.14702 (17)	0.0537 (6)	
H4	0.4250	0.9092	−0.2119	0.064*	
C5	0.3266 (3)	0.9881 (2)	−0.08765 (16)	0.0477 (5)	
H5	0.3662	1.0772	−0.1136	0.057*	
C6	0.2334 (3)	0.9589 (2)	0.01207 (14)	0.0383 (4)	
C7	0.2019 (3)	1.0706 (2)	0.06718 (15)	0.0394 (4)	
H7	0.2356	1.1580	0.0320	0.047*	
C8	0.1020 (3)	1.18087 (19)	0.20795 (14)	0.0358 (4)	
C9	0.1713 (3)	1.3105 (2)	0.16491 (16)	0.0453 (5)	
H9	0.2440	1.3228	0.1023	0.054*	
C10	0.1317 (3)	1.4201 (2)	0.21548 (17)	0.0485 (5)	
H10	0.1778	1.5064	0.1867	0.058*	
C11	0.0245 (3)	1.4028 (2)	0.30831 (18)	0.0482 (5)	
H11	−0.0018	1.4779	0.3414	0.058*	
C12	−0.0441 (3)	1.2757 (2)	0.35271 (16)	0.0433 (5)	
H12	−0.1157	1.2648	0.4157	0.052*	
C13	−0.0057 (3)	1.16326 (19)	0.30263 (14)	0.0344 (4)	
C14	−0.1822 (3)	1.0000 (2)	0.42216 (14)	0.0369 (4)	
H14	−0.2217	1.0734	0.4551	0.044*	
C15	−0.2531 (3)	0.8672 (2)	0.46599 (14)	0.0367 (4)	
C16	−0.3650 (3)	0.8553 (2)	0.56071 (15)	0.0427 (5)	
H16	−0.3912	0.9332	0.5906	0.051*	
C17	−0.4353 (3)	0.7316 (2)	0.60900 (16)	0.0486 (5)	
H17	−0.5095	0.7254	0.6709	0.058*	
C18	−0.3944 (3)	0.6145 (2)	0.56445 (17)	0.0507 (6)	
H18	−0.4384	0.5293	0.5982	0.061*	
C19	−0.2902 (3)	0.6237 (2)	0.47166 (17)	0.0501 (5)	
H19	−0.2670	0.5449	0.4425	0.060*	
C20	−0.2181 (3)	0.7492 (2)	0.41977 (15)	0.0395 (5)	
C1S	0.6113 (4)	0.6064 (3)	0.1772 (3)	0.0922 (10)	
H1S1	0.6377	0.5284	0.2298	0.138*	
H1S2	0.5154	0.5847	0.1411	0.138*	
H1S3	0.5742	0.6855	0.2081	0.138*	
O1S	0.7639 (3)	0.6357 (2)	0.10906 (15)	0.0848 (6)	
H1S	0.8457 (10)	0.6671 (4)	0.1397 (4)	0.127*	

### Atomic displacement parameters (Å^2^)

**Table:**

	*U*^11^	*U*^22^	*U*^33^	*U*^12^	*U*^13^	*U*^23^
Mn1	0.04290 (19)	0.02961 (15)	0.02400 (15)	−0.00218 (12)	0.00435 (11)	−0.00877 (11)
Cl1	0.0695 (4)	0.0623 (4)	0.0640 (4)	0.0191 (3)	−0.0223 (3)	−0.0216 (3)
N1	0.0434 (9)	0.0363 (8)	0.0313 (8)	−0.0010 (7)	−0.0008 (7)	−0.0095 (7)
N2	0.0406 (9)	0.0335 (8)	0.0296 (8)	0.0000 (7)	−0.0012 (7)	−0.0082 (7)
O1	0.0711 (10)	0.0413 (8)	0.0303 (7)	−0.0094 (7)	0.0070 (7)	−0.0124 (6)
O2	0.0673 (10)	0.0460 (8)	0.0445 (8)	−0.0185 (7)	0.0193 (7)	−0.0215 (7)
C1	0.0462 (12)	0.0448 (11)	0.0256 (9)	0.0027 (9)	−0.0009 (8)	−0.0105 (8)
C2	0.0654 (15)	0.0439 (12)	0.0346 (11)	0.0005 (10)	0.0002 (10)	−0.0125 (9)
C3	0.0662 (15)	0.0590 (14)	0.0373 (11)	0.0093 (12)	0.0022 (10)	−0.0224 (11)
C4	0.0579 (14)	0.0696 (16)	0.0329 (11)	−0.0013 (12)	0.0084 (10)	−0.0159 (11)
C5	0.0521 (13)	0.0557 (13)	0.0346 (11)	−0.0075 (10)	0.0048 (9)	−0.0105 (10)
C6	0.0409 (11)	0.0442 (11)	0.0302 (10)	−0.0005 (9)	−0.0001 (8)	−0.0104 (8)
C7	0.0453 (12)	0.0397 (10)	0.0319 (10)	−0.0054 (9)	0.0019 (8)	−0.0062 (8)
C8	0.0445 (11)	0.0316 (9)	0.0318 (10)	0.0000 (8)	−0.0047 (8)	−0.0074 (8)
C9	0.0592 (14)	0.0397 (11)	0.0355 (11)	−0.0066 (10)	0.0010 (10)	−0.0056 (9)
C10	0.0626 (14)	0.0335 (10)	0.0486 (13)	−0.0058 (10)	−0.0052 (11)	−0.0046 (9)
C11	0.0559 (14)	0.0359 (11)	0.0550 (13)	0.0026 (10)	−0.0020 (11)	−0.0175 (10)
C12	0.0477 (12)	0.0402 (11)	0.0426 (12)	0.0033 (9)	0.0008 (9)	−0.0144 (9)
C13	0.0387 (11)	0.0314 (9)	0.0333 (10)	0.0018 (8)	−0.0039 (8)	−0.0080 (8)
C14	0.0405 (11)	0.0390 (10)	0.0318 (10)	0.0024 (8)	0.0004 (8)	−0.0121 (8)
C15	0.0368 (11)	0.0409 (10)	0.0331 (10)	−0.0011 (8)	−0.0009 (8)	−0.0107 (8)
C16	0.0448 (12)	0.0473 (12)	0.0353 (10)	−0.0006 (9)	0.0057 (9)	−0.0131 (9)
C17	0.0493 (13)	0.0600 (14)	0.0351 (11)	−0.0083 (11)	0.0084 (9)	−0.0113 (10)
C18	0.0568 (14)	0.0497 (13)	0.0438 (12)	−0.0178 (11)	0.0059 (10)	−0.0060 (10)
C19	0.0570 (14)	0.0461 (12)	0.0493 (13)	−0.0142 (10)	0.0095 (11)	−0.0192 (10)
C20	0.0409 (11)	0.0437 (11)	0.0345 (10)	−0.0069 (9)	0.0039 (8)	−0.0117 (9)
C1S	0.093 (2)	0.077 (2)	0.100 (2)	−0.0166 (18)	0.029 (2)	−0.0218 (18)
O1S	0.0820 (14)	0.1037 (16)	0.0640 (12)	−0.0195 (12)	0.0008 (11)	−0.0039 (11)

### Geometric parameters (Å, º)

**Table:**

Mn1—O2	1.8868 (14)	C9—C10	1.377 (3)
Mn1—O1	1.9022 (13)	C9—H9	0.9300
Mn1—N1	2.0944 (16)	C10—C11	1.376 (3)
Mn1—N2	2.1047 (15)	C10—H10	0.9300
Mn1—Cl1	2.2277 (7)	C11—C12	1.377 (3)
N1—C7	1.302 (2)	C11—H11	0.9300
N1—C8	1.420 (2)	C12—C13	1.396 (3)
N2—C14	1.303 (2)	C12—H12	0.9300
N2—C13	1.421 (2)	C14—C15	1.429 (3)
O1—C1	1.325 (2)	C14—H14	0.9300
O2—C20	1.321 (2)	C15—C20	1.412 (3)
C1—C2	1.402 (3)	C15—C16	1.415 (3)
C1—C6	1.411 (3)	C16—C17	1.365 (3)
C2—C3	1.376 (3)	C16—H16	0.9300
C2—H2	0.9300	C17—C18	1.395 (3)
C3—C4	1.387 (3)	C17—H17	0.9300
C3—H3	0.9300	C18—C19	1.369 (3)
C4—C5	1.364 (3)	C18—H18	0.9300
C4—H4	0.9300	C19—C20	1.398 (3)
C5—C6	1.409 (3)	C19—H19	0.9300
C5—H5	0.9300	C1S—O1S	1.374 (3)
C6—C7	1.428 (3)	C1S—H1S1	0.9600
C7—H7	0.9300	C1S—H1S2	0.9600
C8—C9	1.396 (3)	C1S—H1S3	0.9600
C8—C13	1.398 (3)	O1S—H1S	0.8725
			
O2—Mn1—O1	91.47 (6)	C10—C9—H9	120.1
O2—Mn1—N1	148.62 (7)	C8—C9—H9	120.1
O1—Mn1—N1	87.98 (6)	C11—C10—C9	120.50 (19)
O2—Mn1—N2	87.70 (6)	C11—C10—H10	119.8
O1—Mn1—N2	148.11 (7)	C9—C10—H10	119.8
N1—Mn1—N2	76.74 (6)	C10—C11—C12	120.75 (19)
O2—Mn1—Cl1	105.95 (6)	C10—C11—H11	119.6
O1—Mn1—Cl1	108.96 (5)	C12—C11—H11	119.6
N1—Mn1—Cl1	103.86 (5)	C11—C12—C13	119.64 (19)
N2—Mn1—Cl1	101.87 (5)	C11—C12—H12	120.2
C7—N1—C8	120.67 (16)	C13—C12—H12	120.2
C7—N1—Mn1	124.46 (13)	C12—C13—C8	119.69 (17)
C8—N1—Mn1	114.81 (12)	C12—C13—N2	125.41 (18)
C14—N2—C13	120.85 (16)	C8—C13—N2	114.90 (16)
C14—N2—Mn1	124.04 (13)	N2—C14—C15	125.47 (18)
C13—N2—Mn1	114.86 (12)	N2—C14—H14	117.3
C1—O1—Mn1	131.49 (12)	C15—C14—H14	117.3
C20—O2—Mn1	130.62 (12)	C20—C15—C16	118.96 (18)
O1—C1—C2	119.31 (18)	C20—C15—C14	123.61 (17)
O1—C1—C6	122.27 (17)	C16—C15—C14	117.43 (17)
C2—C1—C6	118.39 (18)	C17—C16—C15	121.27 (19)
C3—C2—C1	120.6 (2)	C17—C16—H16	119.4
C3—C2—H2	119.7	C15—C16—H16	119.4
C1—C2—H2	119.7	C16—C17—C18	119.27 (19)
C2—C3—C4	121.3 (2)	C16—C17—H17	120.4
C2—C3—H3	119.4	C18—C17—H17	120.4
C4—C3—H3	119.4	C19—C18—C17	120.7 (2)
C5—C4—C3	119.0 (2)	C19—C18—H18	119.6
C5—C4—H4	120.5	C17—C18—H18	119.6
C3—C4—H4	120.5	C18—C19—C20	121.3 (2)
C4—C5—C6	121.6 (2)	C18—C19—H19	119.3
C4—C5—H5	119.2	C20—C19—H19	119.3
C6—C5—H5	119.2	O2—C20—C19	119.40 (18)
C5—C6—C1	119.06 (18)	O2—C20—C15	122.20 (17)
C5—C6—C7	117.27 (18)	C19—C20—C15	118.39 (18)
C1—C6—C7	123.63 (17)	O1S—C1S—H1S1	109.5
N1—C7—C6	125.92 (18)	O1S—C1S—H1S2	109.5
N1—C7—H7	117.0	H1S1—C1S—H1S2	109.5
C6—C7—H7	117.0	O1S—C1S—H1S3	109.5
C9—C8—C13	119.60 (17)	H1S1—C1S—H1S3	109.5
C9—C8—N1	124.94 (18)	H1S2—C1S—H1S3	109.5
C13—C8—N1	115.45 (16)	C1S—O1S—H1S	109.5
C10—C9—C8	119.82 (19)		

### Hydrogen-bond geometry (Å, º)

**Table:**

*D*—H···*A*	*D*—H	H···*A*	*D*···*A*	*D*—H···*A*
O1*S*—H1*S*···O1^i^	0.87	2.19	2.999 (3)	154

Symmetry code: (i) *x*+1, *y*, *z*.
